# Patient Perspectives on Artificial Intelligence in Medical Imaging

**DOI:** 10.2196/67816

**Published:** 2025-07-28

**Authors:** Jeffry Glenning, Lisa Gualtieri

**Affiliations:** 1 Cedars-Sinai Medical Center, 8700 Beverly Blvd, Los Angeles, CA, 90048, United States, 1 5625952473

**Keywords:** artificial intelligence, medical imaging, patient-centered care, patient participation, health equity, medical ethics, digital health, participatory medicine

## Abstract

Artificial intelligence (AI) is reshaping medical imaging with the promise of improved diagnostic accuracy and efficiency. Yet, its ethical and effective adoption depends not only on technical excellence but also on aligning implementation with patient perspectives. This commentary synthesizes emerging research on how patients perceive AI in radiology, expressing cautious optimism, a desire for transparency, and a strong preference for human oversight. Patients consistently view AI as a supportive tool rather than a replacement for clinicians. We argue that centering patient voices is essential to sustaining trust, preserving the human connection in care, and ensuring that AI serves as a truly patient-centered innovation. The path forward requires participatory approaches, ethical safeguards, and transparent communication to ensure that AI enhances, rather than diminishes, the values patients hold most dear.

## Introduction

The integration of artificial intelligence (AI) into medical imaging is widely regarded as a groundbreaking advancement, with the potential to enhance the speed, accuracy, and efficiency of radiological diagnoses [1]. For patients, this can lead to faster results, earlier disease detection, and more personalized treatment plans. In the realm of medical imaging, AI systems represent the next frontier of innovation—building on trends such as outsourcing—by transforming clinical workflows with rapid and highly precise diagnostic capabilities.

However, as AI transitions from experimental stages to clinical implementation, its success depends not only on technical performance but also on patient perception and acceptance. Understanding patient perceptions is critical to adoption, regardless of AI’s technical promise. Medical imaging, such as other areas of health care, depends on both technical expertise and the trust inherent in patient-provider relationships. While AI has demonstrated remarkable accuracy in medical diagnostics, its presence can alter the dynamics of these relationships.

The purpose of this commentary is to synthesize current evidence on patient perspectives regarding AI in medical imaging and to argue that proactively understanding and integrating these views through participatory approaches is indispensable for the successful and ethical adoption of AI in radiology. Here, “successful adoption” is defined not merely by technical performance, but by AI’s ability to enhance diagnostic capabilities while simultaneously building patient trust, ensuring equitable access, and preserving the human-centered nature of care. Without centering patient perspectives, AI risks becoming a technology that, despite its potential, fails to achieve widespread acceptance or deliver its benefits equitably.

## AI in Medical Imaging and Participatory Medicine

AI in medical imaging is rapidly advancing, creating a dual challenge: enhancing diagnostic capabilities while integrating these advancements into patient-centered care. AI systems can process vast amounts of data at unprecedented speeds, offering significant support to radiologists in quickly detecting complex diseases and identifying patterns that may be difficult for even highly trained human eyes to detect [2]. This technological efficiency, however, may not be enough on its own. AI use should be considered within the broader framework of participatory medicine.

Participatory medicine emphasizes patients as active partners in their care. This approach challenges traditional models of health care, where decisions are made for patients rather than with them. In the context of AI, participatory medicine emphasizes the importance of ensuring that patients’ concerns and priorities are integral to the design and deployment of these technologies. Research has revealed that many patients are not fully aware of how AI is integrated into medical imaging, which may hinder its acceptance [3,4]. Without a basic understanding of how AI is used, patients cannot meaningfully participate in decisions about its implementation or provide informed consent for its use. For AI to be successful from the perspectives of all stakeholders, it should strive to be transparent, accessible, and, most importantly, aligned with the values of the patients it serves. A participatory approach could build on successes in other medical domains by establishing patient advisory boards to provide input on AI implementation, collaborating with patients to develop educational materials that explain AI’s role in their care, and offering patients choices regarding the level of AI involvement in their diagnostic process.

## Understanding Patient Perspectives on AI in Medical Imaging

While technical performance is significant, the integration of AI into medical imaging may also be shaped by how patients perceive and accept these technologies [3,5]. A growing body of literature reveals a complex tapestry of patient attitudes, characterized by cautious optimism, specific concerns, and a strong desire for human oversight. Understanding these perspectives is critical for developing and deploying AI in a manner that is not only technologically advanced but also genuinely patient-centered.

## Broad Patient Attitudes Toward AI in Health Care

Establishing patient trust is foundational for the successful integration of AI into medical imaging. This trust is often linked to patients’ understanding of AI’s general role in their care, its potential benefits, and its inherent limitations. While many patients recognize the broad potential of AI to enhance diagnostic accuracy and efficiency across health care, general concerns persist regarding where to appropriately place their trust, alongside fears of diminishing the essential human connection in medical interactions [5]. Patients often approach AI with a blend of hope for improved outcomes and apprehension about the technology’s autonomy and the security of their data [4,5]. This initial disposition underscores the need for clear communication and transparency from the outset.

## Key Themes in Patient Perspectives on AI in Medical Imaging

When focusing specifically on AI in medical imaging, several key themes emerge consistently from patient perspectives.

### Conditional Trust and Human Oversight

A predominant theme is that of conditional trust. While patients are often open to AI, it is typically viewed as a complement to, not a replacement for, human clinical expertise. For example, a study on AI in mammography found that the majority of women surveyed (77.8%) were uncomfortable with the idea of AI functioning independently without radiologist oversight [6]. This sentiment is echoed in other research, with one study finding that 76% of patients would not be comfortable receiving a diagnosis generated solely by AI [7]. The prevailing view is that AI should augment the clinician’s role, supporting human decision-making rather than supplanting it. Central to this is the interaction between clinician and patient—a relationship ideally built on empathy, communication, and trust, qualities AI cannot yet replicate. Patients value the communication they receive from radiologists, reporting that personal interaction enables them to ask questions comfortably and develop a shared understanding of findings [8]. Concerns about a potential lack of human connection are common, with patients emphasizing the importance of human empathy and the “ability to understand with flexibility” [5]. Research consistently indicates a strong patient preference for human involvement in interpreting diagnostic findings, reinforcing the idea that AI is a tool to support, not replace, the human touch that defines patient-centered care [9]. The radiologist’s expertise remains critical in ensuring that AI’s outputs are interpreted and communicated with empathy and clarity.

### Hopes for Enhanced Diagnostic Capabilities and Efficiency

From patients’ perspectives, a significant promise of AI in medical imaging lies in its potential to improve diagnostic accuracy and reduce waiting times [5]. This is not seen as a theoretical gain but as an immediate practical advantage. AI’s ability to rapidly analyze large volumes of data without experiencing human constraints such as fatigue offers advantages in environments where errors can have critical consequences. Research suggests that patients generally have an optimistic outlook regarding AI’s potential to streamline diagnostic workflows [4,7,10]. Many patients hope AI can reduce the anxiety associated with waiting for test outcomes [5]. By expediting image analysis, AI may enable radiologists to communicate results more promptly, thereby reducing psychological distress. In addition, AI systems are sometimes viewed as valuable for providing personalized health information in a timely manner, potentially empowering patients in their health care decisions [4].

### Apprehensions and Ethical Concerns

Despite optimism, patients remain cautious about potential trade-offs. While expecting faster analysis, they also worry about AI’s limitations, fearing it might lead to narrow interpretations or incorrect diagnoses [8]. The quality, trustworthiness, and accuracy of medical information provided by AI systems are major patient concerns [4]. The perception that AI could overlook critical information or misinterpret complex data highlights the need for rigorous validation before clinical integration. A primary apprehension is the potential depersonalization of care, with patients concerned about becoming “numbers” in a technology-driven system [8,10]. This stems from the perception that AI, despite technical proficiency, may lack the emotional intelligence integral to effective care [8]. Furthermore, some patients worry about over-reliance on AI at the expense of human judgment [7,10]. Although generally viewing AI-based systems positively, they often express that such technologies should serve as a supportive tool, reflecting an understanding of AI’s limitations in intuitive and compassionate decision-making [4,5,7,8].

## Demographic Nuances in AI Perception

### Overview

Some studies suggest trends related to age, education, or gender in how patients perceive AI in medical imaging, but these should be interpreted with caution to avoid overgeneralization and amplifying small differences found in small studies. Individual views are paramount, and people are not defined by their demographics. These observations primarily highlight the need for adaptable, person-centered communication rather than rigidly tailored approaches based on demographic profiles.

### Age-Related Differences

Age has been observed to influence patient perceptions. Some studies suggest older adults may exhibit more skepticism toward standalone AI systems, often emphasizing the need for radiologist oversight [3,7-9,11]. Research indicates that older participants (≥65 y) have reported higher concerns regarding AI’s trustworthiness and accountability compared to younger groups [7,10], with notable discomfort regarding personal data security [10]. Furthermore, they have, in some studies, tended to rate AI lower in terms of efficiency, perceiving limited potential for improving health care processes [9,10]. Younger patients, in contrast, have sometimes expressed greater openness to AI integration, highlighting AI’s potential role in enhancing efficiency [7-10], reducing wait times [7,8], and improving access [8]. In some instances, they have displayed confidence in AI-assisted interpretations, demonstrating a readiness to trust AI when validated as accurate and reliable [6-8,11].

### Education and AI Trust

Educational attainment has also been identified as a factor. Some studies indicate that university-educated patients may exhibit higher confidence in AI’s capabilities and express more willingness to accept its use, especially if AI demonstrates superior diagnostic performance [7,9-13]. They may also be more likely to trust hybrid AI-radiologist models and prioritize AI’s ability to enhance precision [3,9]. Conversely, individuals with lower formal educational levels have, in some research, exhibited greater skepticism [7,9,12,13], sometimes viewing AI as a “black box” lacking transparency [8]. They may place greater emphasis on human oversight and radiologist accountability [8].

### Gender-Based Variations

Some studies have reported gender-based differences in AI perceptions. Women have, at times, been found to be more skeptical, voicing concerns about AI’s ability to replace human empathy and judgment [7,10]. They may place greater emphasis on personal interactions with radiologists and express a stronger preference for clinician-led care [8]. Conversely, men have, in some contexts, exhibited greater confidence in AI as a diagnostic tool, particularly when emphasizing efficiency and cost-effectiveness [7,8], and expressed fewer concerns about depersonalization, while still emphasizing the need for clear accountability [8].

### Implications of Observed Variations

While observed trends in some studies suggest that factors such as age, education, or gender may sometimes correlate with varying nuances in AI perception [3,6-13], it is crucial to avoid generalizations and stereotyping. People are individuals, not merely representatives of demographic groups. These observations should sensitize providers to the potential diversity of patient concerns and starting points. The most effective approach is always person-centered: actively listening to each patient, eliciting their specific questions and anxieties, and providing clear, empathetic explanations. For example, focusing on procedural transparency and human oversight may be helpful for any patient expressing skepticism, regardless of age. Similarly, simplifying complex AI concepts can benefit any patient, irrespective of educational background. The goal is to foster trust through responsive, individualized dialog that acknowledges potential differences in starting points or concerns without prejudging individuals based on demographic characteristics.

## The Enduring Imperative of Human Oversight in an AI-Assisted Future

The existing landscape of radiologist-patient interaction is diverse. In certain radiological subspecialties, such as mammography or interventional procedures, direct consultation and the development of ongoing patient-radiologist relationships are relatively common. However, in many other areas of diagnostic radiology, communication is frequently mediated through referring clinicians, meaning patients may have limited or no direct contact with the radiologist responsible for interpreting their medical images. This variability in direct human connection forms a critical backdrop to the introduction and perception of AI in the field.

Perhaps ironically, the integration of AI into medical imaging, a technology often perceived as potentially distancing, appears to intensify, rather than diminish, the patient need for assurance that human experts remain firmly in control and centrally involved in their care [5,8,9]. Even in scenarios where direct patient-radiologist interaction is traditionally low, the knowledge that a skilled human clinician is ultimately responsible for overseeing AI-generated findings, critically evaluating its outputs, and making the final diagnostic decision is paramount for patient trust [5,9]. The perceived “distance” in mediated communication pathways could, in fact, heighten anxieties about AI if this human element—the radiologist’s expertise, ethical responsibility, and ultimate accountability—is not proactively and clearly affirmed. Therefore, as AI tools become more prevalent, the focus should extend beyond simply maintaining existing levels of human interaction; it should actively reinforce and communicate the indispensable role of human clinical judgment in the diagnostic loop. This ensures that patients trust the process and the outcomes, confident that technology serves as an aid to, not a replacement for, human expertise.

## Accountability and Ethical Concerns

As AI takes on a more significant role in medical imaging, questions of accountability become inevitable. Who is responsible if AI contributes to diagnostic errors or adverse outcomes? Studies suggest that patients generally support shared accountability among hospitals, radiologists, and AI developers, reflecting a desire for clarity in how such errors are addressed [13]. Ethical guidelines, including the multisociety statement on AI in radiology endorsed by organizations such as the American College of Radiology and European Society of Radiology, emphasize that ultimate accountability should rest with human clinicians and developers [14,15]. These principles align with patients’ preference for human oversight, which reinforces trust in the health care system.

**Figure 1.**
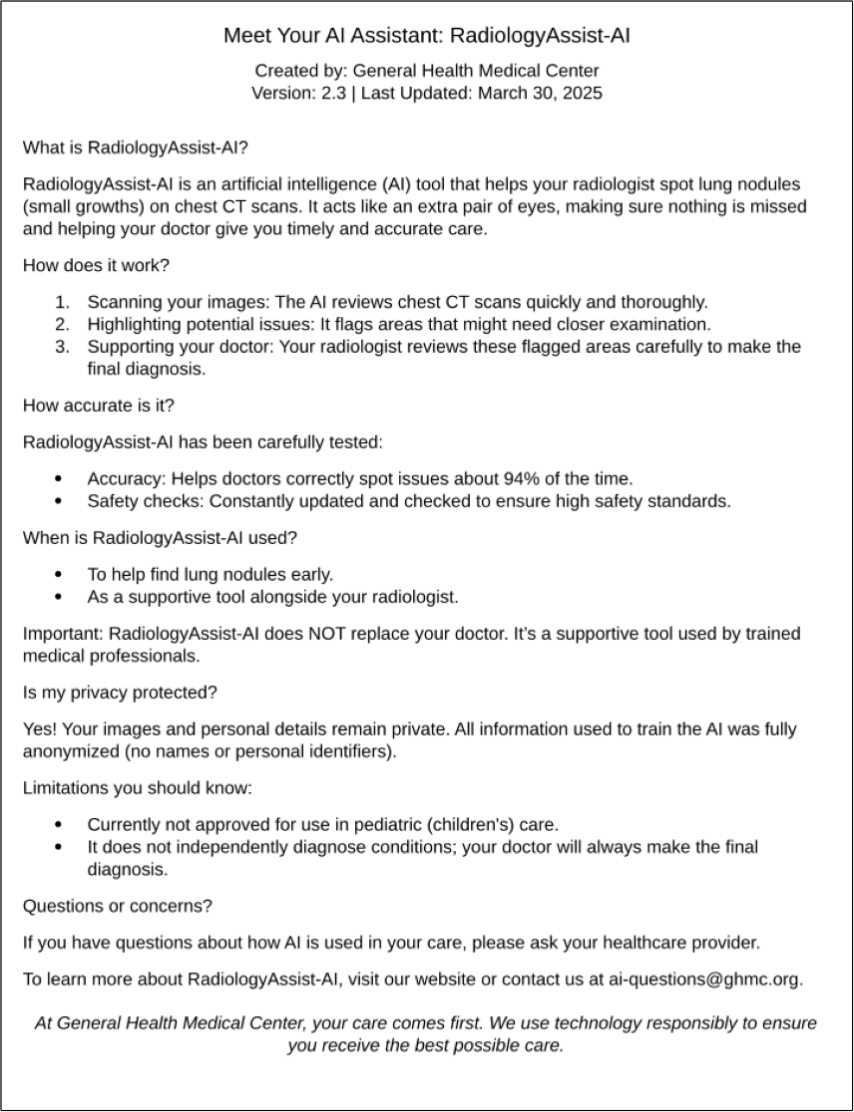
Sample patient-facing model card for AI-supported lung cancer screening.

While questions of accountability are central to ethical concerns, transparency in how AI functions is equally critical in building patient trust. Many patients express interest in understanding the role of AI in their care, including its limitations, accuracy, and potential risks [4,8]. Tools such as “model cards” (Figure 1) have been proposed to outline an AI system’s design, intended use, performance characteristics, and known limitations [16]. Some researchers also advocate for more comprehensive “System Cards” to provide in-depth analyses of AI performance and biases, which could enable clinicians to better explain the technology to patients [15]. However, despite these proposals to enhance transparency by detailing AI design, performance, and biases, the routine integration of such tools into clinical systems for direct patient access or automated sharing is not yet widespread. Consequently, transparency often relies heavily on clinicians to convey this information, underscoring the need for more systemic and readily accessible solutions. Frameworks such as the FDA’s Software as a Medical Device classification may also help clinicians clarify AI’s intended functions—whether assisting with measurements, highlighting abnormalities, or offering diagnostic suggestions—ensuring that patients have a clearer understanding of the technology’s role in their care [16].

## Transparency in AI Deployment

Achieving transparency in the deployment of AI, which is crucial for fostering patient confidence and engagement, is a shared responsibility. While individual clinicians are at the frontline of patient communication, health care organizations hold a fundamental responsibility for establishing policies, ethical frameworks, and technological infrastructures that mandate and support transparency regarding AI use. This includes providing clinicians with the necessary training and tools [15]. With this foundational support, transparency becomes particularly important in clinical decision-making, where AI’s role should be clearly communicated to support trust and engagement. When AI plays a significant role in shaping diagnostic or treatment recommendations, clinicians could consider including information in imaging reports or after-visit summaries about:

1. The specific role AI played in the diagnostic process (eg, prioritizing findings, generating a differential diagnosis, or suggesting treatment pathways).
2. How the clinician evaluated and incorporated AI recommendations into their final decision.
3. Whether the AI’s recommendation differed from the clinician’s judgment and, if so, the reasoning behind the chosen course of action.

In instances where AI and clinician recommendations diverge, it is crucial to clarify the context of such divergence. This pertains to scenarios within the clinical diagnostic process where an AI tool used by the health care team generates a finding or recommendation that differs from the supervising clinician’s independent assessment. It does not primarily refer to patients independently consulting consumer-facing AI tools, an emerging area with its own distinct considerations. The focus here is on how clinicians navigate these situations and transparently communicate decisions when their expert judgment and an AI’s output are not fully aligned. Shared decision-making discussions with patients are then vital to ensure transparency and respect for patient autonomy. These discussions should include explaining the differences between the AI’s recommendation and the clinician’s judgment, the rationale for the clinician’s chosen course of action, and an affirmation that the clinician’s expertise ultimately guides the final decision-making process.

Patients may also benefit from understanding the validation status and certification of the AI systems involved in their care. This could include sharing whether the AI system has been approved by regulatory bodies, such as the FDA or equivalent agencies [16], and any information on its intended use and known limitations. Including these details in patient education materials or as part of prediagnostic consent processes might help demystify the technology and enhance patient confidence in its use.

## Data Privacy and Security

Data privacy and security concerns remain a central issue for patients, many of whom are willing to share their health data for AI development only if robust protections are in place [4,5]. Legal frameworks such as General Data Protection Regulation (GDPR) in Europe and Health Insurance Portability and Accountability Act (HIPAA) in the United States establish foundational safeguards, while advanced methods such as differential privacy and federated learning are being explored to address privacy concerns specific to AI systems [17]. Offering patients clear information about their rights regarding data inclusion, such as the ability to opt out even for anonymized datasets, may further demonstrate respect for their autonomy and foster trust [15]. Ensuring that patients feel informed and protected is likely to play a critical role in their acceptance of AI.

Ensuring that patients feel informed and protected regarding their data is likely to play a critical role in their acceptance of AI. Recognizing the multifaceted ethical challenges, including those related to data governance and transparency, prominent national and international radiology societies such as the American College of Radiology, Canadian Association of Radiologists, European Society of Radiology, Royal Australian and New Zealand College of Radiologists, and Radiological Society of North America ( are actively developing guidelines and practical recommendations for the ethical development, deployment, and monitoring of AI tools [15]. These comprehensive efforts include emphasizing the need for robust data privacy measures, clear data handling protocols, and continuous education for radiologists on AI’s capabilities and limitations to help manage biases and challenges related to AI systems [15]. Open communication about AI’s limitations, alongside reassurance of ongoing clinician involvement, can further address patient concerns and foster trust in AI-supported care.

By addressing these ethical considerations with a patient-centered approach, health care providers can better align AI implementation with patient expectations. Transparency, accountability, and proactive engagement with patient concerns are essential for fostering trust and ensuring that AI in medical imaging ultimately enhances the quality of care and patient outcomes.

## Algorithmic Bias, Health Disparities, and the Erosion of Patient Trust

The efficacy and fairness of AI systems in medical imaging are fundamentally dependent on the data they are trained on. The use of unrepresentative datasets in AI training not only risks developing algorithms that perform inequitably across diverse patient populations [18], thereby potentially leading to misdiagnosis and exacerbating existing health disparities, but it can also severely undermine patient trust. If patients, particularly from underrepresented or historically marginalized groups, perceive or learn that AI systems may not be accurate or fair for them, their confidence in AI-assisted diagnostics—and potentially the health care system using them—will inevitably be eroded.

Demographic imbalances in training datasets—whether related to race, ethnicity, gender, age, or socioeconomic status—can introduce insidious biases into AI models. An AI system predominantly trained on images from one demographic group may exhibit reduced accuracy or reliability when applied to others, leading to diagnostic errors or missed conditions for those in underrepresented groups [18]. This not only perpetuates but can amplify existing health inequities. From a patient perspective, the realization that an AI tool might be less effective or even harmful due to their demographic background strikes at the core of equitable care and can foster deep-seated mistrust.

Therefore, ensuring that AI models are developed and validated using diverse, representative datasets is not merely a technical imperative but a crucial ethical obligation directly linked to patient well-being and trust. Proactive strategies to detect, assess, and mitigate bias throughout the AI lifecycle are essential [18]. This commitment to fairness and equity is fundamental to building AI systems that are genuinely transparent, trustworthy, and capable of enhancing health care for all patients, thereby upholding the principles of participatory and patient-centered medicine.

## Balancing Systemic Benefits and Risks of AI in Medical Imaging

The integration of AI into medical imaging offers a complex interplay of substantial potential benefits and notable risks that extend beyond immediate patient perceptions, impacting clinical workflows, health care systems, and the practice of radiology itself.

### Systemic and Operational Benefits

Beyond the enhancements to diagnostic accuracy and efficiency that are often highlighted, AI presents several broader advantages:

1. Standardization and quality improvement: AI tools can contribute to greater consistency in image interpretation and reporting, potentially reducing inter-reader variability and supporting adherence to best-practice guidelines [1,15].
2. Workflow optimization and radiologist support: AI can automate repetitive or time-consuming tasks (eg, image segmentation and preliminary flagging of normal studies), prioritize urgent cases for review, and serve as a “second reader,” potentially alleviating radiologist workload, reducing burnout, and allowing more focused attention on complex cases or direct patient communication where appropriate [1,15].
3. Advancement of medical knowledge: the application of AI to large-scale imaging datasets can accelerate research, facilitating the discovery of novel imaging biomarkers, improving understanding of disease pathophysiology, and aiding in the development of personalized medicine approaches [2,15].
4. Potential for enhanced accessibility: in resource-constrained environments, AI could theoretically augment diagnostic capabilities where specialist radiologists are scarce, although equitable access and implementation remain significant global challenges [15,16].

### Systemic Risks and Implementation Challenges

Alongside these benefits, a range of risks and challenges should be proactively addressed for responsible AI adoption:

1. Technical limitations and generalizability: AI models can exhibit “brittleness,” performing well on data similar to their training sets but potentially failing or underperforming when encountering out-of-distribution data, novel disease presentations, or images from different scanners or protocols. Ensuring robustness and reliable generalization across diverse clinical scenarios is a critical ongoing challenge [15,18].
2. Automation bias and clinician over-reliance: a significant concern is the potential for ‘automation bias,’ where clinicians may develop an undue reliance on AI-generated outputs, potentially accepting incorrect AI suggestions without sufficient critical scrutiny, or experiencing a gradual deskilling in certain interpretive tasks. This can diminish the vital role of human judgment and oversight, potentially leading to diagnostic errors if AI outputs are not rigorously evaluated as part of a comprehensive clinical assessment [7,13].
3. Integration and workflow disruption: successfully embedding AI tools into established clinical workflows is a complex undertaking, often requiring substantial investment in IT infrastructure, interoperability solutions, staff training, and careful redesign of existing processes to avoid unintended negative consequences. This challenge of workflow disruption with new technology is not unique to AI, as similar significant issues have been well documented with the integration of electronic health records [19].
4. Data governance, privacy, and algorithmic bias: ensuring robust data governance, protecting patient privacy, and actively mitigating algorithmic biases that could exacerbate health disparities are fundamental prerequisites for ethical AI deployment [17,18].
5. Interpretability and the “black box” issue: the lack of transparency in the decision-making processes of some complex AI models (the ‘black box’ phenomenon) can pose challenges for clinical validation, error analysis, establishing clinician trust, and explaining AI-influenced decisions to patients.
6. Regulatory, legal, and ethical frameworks: the evolving regulatory landscape for AI as a medical device, along with establishing clear lines of accountability for AI-related errors and navigating other ethical complexities, requires ongoing attention and development of robust governance structures [14-16].

Effectively harnessing AI’s transformative potential in medical imaging necessitates a comprehensive strategy that actively seeks to maximize these benefits while diligently mitigating the associated risks through rigorous validation, continuous performance monitoring, comprehensive clinician training, and transparent, adaptive governance frameworks.

## Recommendations for Patient-Centered AI Integration

The successful and ethical integration of AI into medical imaging is contingent upon addressing core patient expectations, primarily the need for transparency regarding AI’s role and the paramount importance of preserving human interaction and oversight. These foundational patient priorities necessitate proactive, concrete strategies to ensure AI adoption is patient-centered and builds trust; the following recommendations aim to guide this process.

### Enhance Transparency and Build Trust

Patients consistently express a desire to know whether AI contributed to their diagnosis and the specific role it played [4,5,8].

- Clear communication: provide clear and accessible information about AI’s involvement, such as labeling AI-assisted results in medical records or patient portals. This demystifies the technology and empowers patients for informed discussions.
- Implement transparency tools: the “Model Cards” (Figure 1) [16] and “System Cards” [15] previously discussed offer structured ways to detail AI design, performance, and limitations. These tools should be actively pursued and integrated into clinical practice. Doing so can empower clinicians in their discussions with patients and significantly support informed consent processes.

- Shared decision-making: incorporate AI into shared decision-making processes, allowing radiologists to explain how AI contributed to a diagnosis and discuss how its outputs align with clinical observations. This fosters transparency, trust, and patient empowerment.

### Uphold the Primacy of Human Interaction and Empathy

While patients appreciate AI’s ability to enhance diagnostic accuracy and efficiency, they emphasize the irreplaceable value of human empathy and clinical judgment [5,8,9].

- Reinforce clinician role: radiologists play a critical role in interpreting AI-generated results and ensuring that these insights are communicated with clarity and compassion. AI should be consistently framed as a tool to support, not replace, the human connection that underpins trust and comfort in healthcare settings.

### Champion Participatory Approaches

Achieving meaningful patient engagement requires actively involving patients in the development, deployment, and evaluation of AI technologies.

- Patient advisory boards: establish advisory boards composed of diverse patient representatives to ensure patient concerns and priorities are integrated into decisions about AI development and implementation, including input on algorithm design, ethical guidelines, and clinical workflows.
- Cocreation of educational materials: collaborate with patients to create accessible materials (eg, visual guides, videos, and interactive platforms) that explain AI’s capabilities, limitations, and role in imaging, tailored to different patient populations.
- Feedback mechanisms: develop channels for patients to provide feedback on their experiences with AI-driven diagnostics to help refine these systems and ensure they meet patient needs and expectations.

### Ensure Ethical Governance and Accountability

Ethical considerations are crucial for patient acceptance and trust.

- Clear accountability: establish and communicate clear lines of responsibility among radiologists, AI developers, and health care institutions in the event of diagnostic errors involving AI.
- Data privacy and security: maintain robust safeguards for sensitive patient information. Transparent communication about data use and compliance with privacy regulations (eg, HIPAA and GDPR) is essential to reinforce patient trust.
- Mitigate bias: proactively address and mitigate potential biases in AI algorithms (as discussed in “Algorithmic Bias, Health Disparities, and the Erosion of Patient Trust”) to ensure equitable outcomes.

### Foster Patient Agency and Continuous Improvement

Looking ahead, empower patients and ensure AI systems evolve responsibly.

- Promote patient choice: as AI technologies become more transparent and validated, explore offering patients understandable options regarding AI tools or diagnostic pathways, where clinically appropriate and feasible, to enhance autonomy.
- Incorporate patient feedback for iteration: use patient feedback to continuously improve AI systems, ensuring they remain responsive, ethical, and centered on patient needs.

Meeting patient expectations for AI in medical imaging requires more than technological advancement. It demands a thoughtful, inclusive approach that prioritizes transparency, human connection, participatory engagement, and ethical integrity. By addressing these priorities, AI can enhance clinical workflows, support equity, and improve the patient experience, ensuring its transformative potential benefits all.

## Future Directions

### Overview

The integration of AI into medical imaging is a dynamic and evolving field. Continued vigilance and proactive adaptation focused on patient-centered principles will be essential for its responsible advancement and to realize its full transformative potential, moving beyond mere enhancement of current practices. To guide this evolution and address remaining knowledge gaps, several key research and development priorities emerge.

### Enhancing Patient Education and Meaningful Understanding

Future efforts should go beyond basic information provision. Research should focus on developing and rigorously evaluating innovative educational strategies that effectively clarify AI’s role, capabilities, and inherent limitations in medical imaging. The goal is to foster genuine, informed trust and empower patients to engage meaningfully in discussions about AI-assisted care, moving past potential skepticism or uncritical acceptance [9].

### Longitudinal and Cross-Cultural Assessment of Patient Attitudes

As AI becomes more deeply embedded in clinical practice, it is crucial to conduct longitudinal studies. These studies should track the evolution of patient attitudes, concerns, and expectations over time. Furthermore, comparative research across diverse health care systems and cultural contexts is needed to understand how varying societal values and health care structures influence patient perspectives on AI.

### Optimizing Clinician-Patient Dynamics in AI-Mediated Care

The impact of AI integration on radiologist-patient relationships and communication warrants deeper investigation. Research should explore how AI-enabled tools—such as interactive reports or AI-augmented consultation platforms—can be designed to enhance, rather than hinder, patient trust, comprehension, and engagement, particularly in varied communication models (direct vs mediated).

### Strengthening Ethical Frameworks and Championing Patient Co-Design

The ethical governance of AI requires continuous refinement, with an unwavering focus on accountability, data security, privacy, and the mitigation of bias. Critically, future research and development should prioritize the direct and active involvement of patients as collaborators and co-designers throughout the AI lifecycle—from conceptualization and algorithm development to deployment and evaluation—to ensure AI solutions truly align with the “nothing about me without me” principle.

## Conclusions

AI holds transformative potential for medical imaging, promising enhancements in diagnostic accuracy, efficiency, and patient outcomes. However, this commentary has argued that the successful and ethical realization of this potential is inextricably linked to a patient-centered approach. Such an approach should prioritize transparency, uphold the critical role of human connection and oversight, and actively integrate patient perspectives through participatory methods.

While patients express conditional optimism toward AI, their trust is contingent upon addressing concerns regarding depersonalization, accountability, fairness, and data privacy. As demonstrated, fostering this trust requires more than technological sophistication; it demands a commitment to clear communication, shared decision-making, and the cocreation of AI solutions with patients, not just for them.

Ultimately, the integration of AI into medical imaging will be most beneficial if it reinforces, rather than erodes, the humanistic core of health care. By embracing the principles of participatory medicine, stakeholders—radiologists, developers, institutions, and policymakers—can collaboratively guide AI’s evolution. This ensures that AI serves as a tool to empower individuals, reduce health disparities, and elevate the standard of care, truly aligning technological advancement with the enduring values of patient-centeredness and ethical integrity.

## Abbreviations

AI: artificial intelligence
GDPR: General Data Protection Regulation
HIPAA: Health Insurance Portability and Accountability Act
RANZCR: Royal Australian and New Zealand College of Radiologists
RSNA: Radiological Society of North America
